# Online Mindfulness Intervention, Mental Health and Attentional Abilities: A Randomized Controlled Trial in University Students During COVID-19 Lockdown

**DOI:** 10.3389/fpsyg.2022.889807

**Published:** 2022-07-07

**Authors:** Louise Devillers-Réolon, Nicolas Mascret, Rita Sleimen-Malkoun

**Affiliations:** Aix-Marseille University, CNRS, ISM, Marseille, France

**Keywords:** mindfulness meditation, COVID-19 lockdown, mental health, attentional abilities, online practice, university students

## Abstract

The COVID-19 pandemic has led to worldwide restrictive measures, raising concerns about mental health in young adults who were not particularly vulnerable to the virus itself. This study investigated the impact of these restrictions on mental and cognitive health of university students, and tested the efficacy of a brief online mindfulness meditation intervention in countering psychological distress and improving attentional abilities. Ninety-six university students forced into remote learning due to COVID-19 pandemic restrictions and with no experience in meditation were randomly assigned to either a passive control group (*n* = 48) or to an experimental group (*n* = 48) following daily, for 17 days, an online mindfulness intervention (10–20 min per day). Due to drop-out, 38 participants in each group were finally analyzed. Pre- and post-tests assessed participants’ mental health (psychological well-being, depression, anxiety, stress) and attentional abilities. The analysis of baseline data in comparison with normative scores and pre-pandemic statistics confirmed the expected psychological distress, but it did not reveal any attentional deficits in our participants. Pre-post change scores analyses showed a reduction in stress (*p* = 0.006, *η_p_*^2^ = 0.10), anxiety (*p* = 0.002, *η_p_*^2^ = 0.13), and depression (*p* = 0.025, *η_p_*^2^ = 0.07), and an improvement in well-being (*p* = 0.013, *η_p_*^2^ = 0.12) in the experimental group, but not in the control group. In both groups, no significant effect was found on attentional abilities. Our results confirmed the psychological vulnerability of higher education students in the midst of the remote learning period during the second COVID-19 lockdown in France, while suggesting preservation of attentional functioning. Although the tested mindfulness intervention did not enhance the attentional abilities in already good performing students, it did promote their mental health. This study offers additional evidence on the feasibility and efficacy of mindfulness-based interventions in students during psychologically straining periods, like the COVID-19 pandemic.

## Introduction

The COVID-19 pandemic has led to the adoption of unprecedented restrictive measures throughout the world. In France, to the date of the present study, two successive nationwide lockdowns have been imposed: from the 17th of March to the 11th of May 2020, and from the 30th of October to the 15th of December 2020. During these lockdowns, remote working and learning were the rule, with an imposed physical distancing unavoidably leading to social distancing. Such social isolation is known to be one of the key factors that could lead to increased mental health concerns (Killgore et al., 2020), especially in university students who are not particularly vulnerable to the virus itself (Hamza et al., 2021).

An online survey conducted by the French National Public Health Agency on 2000 adults (French Public Health Agency, 2021) showed the psychological impact of the pandemic on mental health. It revealed that at the beginning of the second lockdown, the prevalence of anxiety states increased (20.8% vs. 13.5% in 2017), and the prevalence of depressive states doubled (20.6% vs. 9.8% in 2017) as compared to outside the pandemic. This same survey found that, among the surveyed age groups, the 18–34 years age group was the most psychologically impacted with 28.25% of this population affected by depressive states (vs. 22.5% for 35–49 years, 18.5% for 50–64 years, 14.4% for 65 years and more) and 26.9% affected by anxious states (vs. 23% for 35–49 years, 17.5% for 50–64 years, 14.3% for 65 years and more). The psychological vulnerability of French university students in the midst of the COVID-19 pandemic was also confirmed by a separate study reporting high levels of stress and anxiety in this population (Husky et al., 2020). Interestingly, such high prevalence of mental distress in the younger adults group was not present in pre-COVID-19 published data (Léon et al., 2018), with the 18–24 years age group and the 25–34 years found to be less impacted (15.1% and 14.0% respectively) than the 35–44 years (15.6%). Similar findings highlighting the psychological vulnerability of university students in the midst of the pandemic were also reported in many other countries over the world (e.g., Jordan: Al-Tammemi et al., 2020; Ethiopia: Aylie et al., 2020; Pakistan: Salman et al., 2020; United States: Son et al., 2020).

Knowing the close relationship between emotions and cognitive processes (Tyng et al., 2017), and the effects of changing learning environment and habits on cognitive abilities and academic performance (Shamaki, 2015; Di Pietro et al., 2020), we could expect pandemic-related restrictions to induce cognitive difficulties in students. One key link to learning and academic achievement is attention. It is in fact not possible to understand, learn, or remember something that we do not attend to. Both in-person and online learning situations require goal-directed focused and sustained attention, which can be impaired by anxiety, depression, and stress (Eysenck et al., 2007; Tyng et al., 2017; Keller et al., 2019). Emotions play also an important role in selective information processing through the neural connectivity between the amygdala and the prefrontal cortex (Pessoa and Adolphs, 2010). Therefore, although it was not yet experimentally shown in the context of the current pandemic, we expected that the aforementioned psychological negative impacts of the COVID-19 restrictions on university students would go hand in hand with attentional deficits. Altogether, this could seriously compromise students’ mental health, as well as hinder their academic progress. Although this latter aspect was not directly assessed in our study, we know from previous research that stress (Oduwaiye et al., 2017; Cibrian-Llanderal et al., 2018), depression (Muhammed et al., 2018; Awadalla et al., 2020), and anxiety (Vitasari et al., 2010; Awadalla et al., 2020) are risk factors for poor academic performance or even failure.

Given the seriousness of mental health threat and the potential cognitive difficulties that could be posed by this ongoing pandemic (or any future similar crisis), there is an urgent need to identify preventive and interventional strategies. Indeed, countering such collateral consequences is not trivial and should be a global priority. To this purpose, we tested in this study a non-pharmacological self-management solution based on mindfulness meditation (MM), which can be used as prevention or treatment strategy. MM is a secular mental practice originating from Buddhism. It consists in training one’s attention to be fully drawn to the immediate moment with a sense of curiosity, openness, and acceptance (Bishop et al., 2004; Kabat-Zinn, 2017), or said differently, observing one’s sensations and experiences with equal receptivity regardless of their affective content, without trying to retain the pleasant ones and/or reject the unpleasant ones. MM practice aims at developing the trait of mindfulness, that is a self-awareness of the present experience, including one’s thoughts, feelings, and sensations, without any judgment, filter, or expectations (Bishop et al., 2004; Kabat-Zinn, 2017). Accordingly, by developing a non-elaborative and non-reactive awareness, the meditator supposedly becomes an observer of their own mental activity and interactions with the world resulting in reduced automatic responses (Maran et al., 2021).

MM can be practiced face-to-face or online through smartphone application or audio recordings. Such interventions are feasible in university students, as reported by previous studies using subjective measures like self-reported adherence, outside the particular context of a pandemic (Cavanagh et al., 2018; Danilewitz et al., 2018). Similar reports during COVID-19 are still lacking, except one promising study in first-year psychology students concluding that MM online interventions may be pathway to buffer the mental health burden derived from the COVID-19 pandemic (González-garcía and López, 2021).

During MM practice, the meditator develops an attitude of decentering and non-attachment, resulting in a reduction or even suppression of the normative relationship between internal experiences (e.g., thoughts) and other internal experiences (e.g., sad feelings) or between internal experiences and resulting behaviors (e.g., anger and aggressiveness; Levin et al., 2015). This attitude also lead to a reduced reactivity to unpleasant hedonic tone (Hadash, 2016). The development of this tempered attitude fosters the acceptance of emotions and facilitates their regulation (Vago and Silbersweig, 2012; Malinowski, 2013). MM practice has positive impacts on mental health (for reviews see Keng et al., 2011; Spijkerman et al., 2016). Empirically, MM practice has been found to reduce depression, stress and anxiety, and enhance psychological well-being and positive affect in the general population (e.g., Shapiro et al., 2005; Grossman et al., 2010; Josefsson et al., 2011) and in students (e.g., Ratanasiripong et al., 2015; Zollars et al., 2019), even when practiced online (e.g., Tkatch et al., 2017; Cavanagh et al., 2018; Walsh et al., 2019). In the context of COVID-19 pandemic, encouraging results have been found regarding the psychological benefits of online MM in general population (Green et al., 2021; Khandelwal, 2020; Lim et al., 2020; Matiz et al., 2020; Sanilevici et al., 2021; Zhang et al., 2021). In students, a recent study showed the feasibility of a brief online Mindfulness and Compassion-based Intervention and highlighted its benefits on stress, anxiety and self-compassion (González-garcía and López, 2021). It was conducted with only first-year psychology students during the first COVID-19 lockdown in Spain without including however a control group. Hence, it is not yet demonstrated if an online MM practice can help higher education students to cope with psychological difficulties due to the COVID-19 pandemic, or a similarly mentally straining period that adds to the usual stress faced by students.

On the cognitive level, the consequences of the pandemic on the attentional abilities of university students are not yet established. Furthermore, no evidence exists on the benefits of MM practice on attentional abilities in this context, neither in the general population nor in the higher education students who had to cope with the pandemic-related restrictions including remote learning. Nevertheless, the aforementioned interdependence of emotions and cognitive processes (Tyng et al., 2017) may suggest that mentally distressed students could also be exposed to attentional deficits. Current literature is supportive of positive short and long-term effects of MM practice on cognitive functions (for reviews see Chiesa et al., 2011; Gallant, 2016; Cásedas et al., 2019). Knowing that the two main components of mindfulness are orientation to experience and self-regulation of attention (Bishop et al., 2004; Lutz et al., 2008), attentional abilities are considered to play a central role in MM (Bishop et al., 2004; Malinowski, 2013). They have also been repeatedly shown to improve with MM practice, both in the general population (e.g., Moore and Malinowski, 2009) and in students (Ching et al., 2015), even after a short intervention (e.g., Zeidan et al., 2010; Norris et al., 2018). These attentional benefits were also observed with online MM practice (Spadaro and Hunker, 2016; Walsh et al., 2019). Accordingly, we contend that an online MM practice would help students cope with their potential attentional difficulties during the remote learning period imposed by the COVID-19 pandemic.

Therefore, the overarching objective of this study was to investigate the effects of a brief online MM intervention (MMI) on the mental health and attentional abilities of university students during the lockdown and the remote learning period imposed by the restrictions related to the COVID-19 pandemic. To this end, during the second French lockdown (end of 2020), we compared an experimental group of university students that completed a brief online MMI to a passive control group of the same population on different psychological and attentional measures that were collected at baseline and after the end of the intervention. The MMI was managed through the University online platform, and consisted of guided meditation sessions that were provided through audio recordings. Their content was selected from conventional MM exercises to target the components that were most relevant to the difficulties that university students faced during the studied COVID-19 period (see details in the Materials and Methods section). We objectively measured the adherence of the MM group to the intervention in order to assess the feasibility and suitability of the proposed intervention. In the psychological domain, we were interested in stress, anxiety, depression, and well-being. In the cognitive domain, we were interested in attentional processing and concentration capacities. Compared to outside the pandemic, we expected to observe poor mental health and reduced attentional abilities in our cohort at baseline (i.e., during the remote learning period imposed by the pandemic crisis). Furthermore, we expected the MMI to enhance the mental health and eventually the attentional abilities of the experimental group, relative to the passive control group participants.

## Materials and Methods

### Participants

One hundred volunteers were assessed for eligibility. They were recruited through e-mails and social media platforms (e.g., Facebook) among the students of the host institution. They were university students in their second or third undergraduate years or first graduate year. The inclusion criteria were: (i) actively undertaking a university degree that was switched to remote learning (fully or ≥3 days per week) for at least the past 4 weeks and the coming 4 weeks, due to governmental restrictions imposed to face the COVID-19 pandemic, and (ii) not having previous or current experience in MM. Prior to their enrollment, all participants were given detailed written information about the study, without stating its precise objective or the underlying hypotheses. They all gave their written informed consent to the experimental procedure that agreed with the Declaration of Helsinki and was approved by the Ethics Committee for Research in Science and Techniques of Physical and Sports Activities (CER STAPS no IRB00012476-2020-25-11-70).

**Figure 1:**
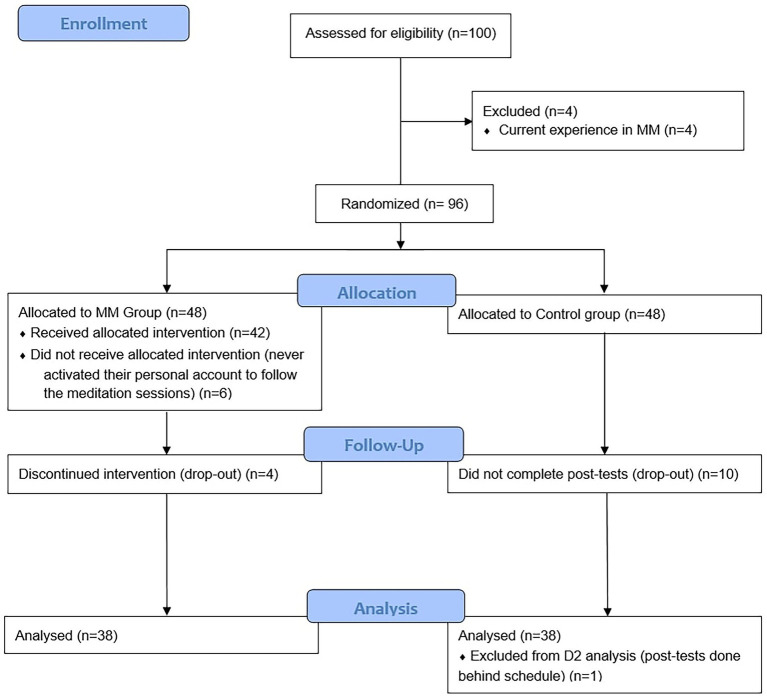
Consolidated Standards of Reporting Trials (CONSORT) flow diagram.

After the exclusion of four participants who have been already practicing MM, the remaining 96 students were randomly allocated to the passive control group (*n* = 48), or to the MM group who followed the online MMI (*n* = 48). Specifically, a non-stratified randomization was conducted *via* an algorithm implemented in MATLAB R2018b (MathWorks, Natick, MA, United States). In the MM group, six participants never activated their personal account to follow MM sessions and four participants dropped-out during the intervention. All six did not complete the post-tests. In the control group, 10 participants did not connect to complete the post-tests. Finally, due to drop-out, complete results (pre- and post-tests) were obtained for 38 participants in the MM group (23 woman, *mean* ± *SD*: 22.43 ± 2.44 years) and 38 in the control group (12 women, *mean* ± *SD*: 21.83 ± 4.13 years), with one participant of the control group excluded from the cognitive analysis as their cognitive test was not completed in due time. The Consolidated Standards of Reporting Trials (CONSORT) flow diagram of the study can be found in Figure 1.

### Procedures

The present experiment was fully conducted online and managed through the secured digital workspace platform of the host University. It lasted 17 consecutive days during the second French lockdown due to the COVID-19 pandemic and before Christmas holidays, from the 30th of November 2020 to the 16th of December 2020. Only the last 2 days were out of lockdown. However, an imposed curfew between 8 pm and 6 am remained, and there was no change in the remote learning obligation for university students. Participants were randomly assigned to a control group with no specific intervention or an experimental group (MM group) following the online MMI.

#### Assessment

All participants underwent a remote psychological (stress, depression, anxiety, well-being) and cognitive (attentional abilities) assessment at the beginning (pre-tests) and the end (post-tests) of the study. For both control and experimental groups, the pre- and post-tests were phased, respectively, during the 2 days preceding and following the MMI, ensuring that participants completed their post-tests before Christmas holidays and upcoming potential national alleviation of COVID-19 restrictions. The day before the pre- and post-tests, the participants were reminded by e-mail of their individual assessment time slot and the general requirements for completing the tests: seated comfortably, alone in a quiet room away from distractions, and using the same computer equipment in post- and pre-tests. On the day of the planned test, the participants received an e-mail with their individual internet links that allowed them to complete their tests at the fixed time.

Participants of the experimental group followed their daily mindfulness intervention, after logging in with their personal username and password, which allowed us to keep a daily log of their participation. They also completed a short survey just after their meditation session evaluating the extent to which they managed to follow the intervention.

#### Interventions

Participants of the control group were instructed to continue with their normal daily activities. Participants of the MM group were proposed a daily online mindfulness practice session which lasted around 10 min on the first day and during the weekend (Sunday and Saturday), and around 20 min during the week (from Monday to Friday), over a 17-day period. On the first day of the MMI, an introductory meeting with the experimental group was organized *via* the videoconferencing platform Zoom using the University license, followed by the streaming of the first online guided-mindfulness meditation session. The following days, the audio recording was posted on the secured online page of the study hosted by the University’s digital workspace platform with exclusive access to the members of the MM group. After logging in, the participants played the recording providing them with the general instructions followed by the MM exercise of the day. At the end of the session, participants were invited to grade how well they managed to follow the session. The daily recording was available from 6 pm to 11 pm hence allowing each participant to login and follow the meditation practice at their convenience.

The recordings and their content were chosen and provided by a meditation instructor who trained in several meditative practices including Mindfulness-Based Stress Reduction (MBSR) and Vipassana. He has 6 years of experience in online guided mindfulness meditation practice through a mobile application he founded and through which he collaborates with the director of the Mindful France Institute, a psychiatrist trained in mindfulness-based therapies. All of the sessions lasted around 10 or 20 min and followed a similar structure: a short introduction, general instructions (posture, distractions, focus on the breath), and then the proposed exercise followed by a phase-out conclusion with general recommendations until the next session. Sessions addressed three main topics chosen in accordance with the reported psychological difficulties encountered by students during the COVID-19 pandemic: (i) the consciousness of breathing, (ii) the development of positive emotions, and (iii) the development of acceptance. Breathing exercises, inspired by fundamental MBSR programs, were chosen to initiate to the practice. In breathing exercises, attention is focused on the sensation of the breath to avoid distractions and redirect awareness in a non-reactive and non-elaborative manner to the present moment. In positive emotions exercises, visualization is used to consciously orient thoughts toward enthusiasm, hope, and happiness-inducing situations. Acceptance exercises aimed at learning how to better tolerate pain, difficulties, negative emotions, and sensations, without judging, avoiding and/or amplifying them. The detailed content and organization of all the sessions are reported in Supplementary Table 1.

The participants who did not connect to the University’s digital workspace platform or forgot to complete the daily survey were contacted the next day to remind them of the importance of regular practice and the completion of the post-meditation survey, as well as to encourage them to participate in the upcoming sessions. Furthermore, all participants received regular motivational newsletter e-mails including links to short videos or texts with general information about meditation and mindfulness-inspired recommendations and quotes.

### Measures

#### Adherence to the MMI

We first measured the completion rate of the MMI, i.e., the percentage of participants of the MM group who completed the pre- and post-tests. Secondly, the adherence rate to MM sessions was objectively measured through the connections log to the University’s digital workspace platform and the completion of the daily psychological questionnaires and/or the survey. The content of the survey was not analyzed. It was only used to ensure that the participants had followed the MM session.

#### Mental Health

##### Stress, Anxiety, and Depression

The French version (Donald, 2012) of the Depression Anxiety Scale 21 (DASS21; Henry and Crawford, 2005), derived from the DASS42 (Lovibond and Lovibond, 1995) was used to assess stress, anxiety, and depression severity during the past week. Seven items were used to assess self-reported stress (e.g., “*I found it hard to wind down*”), seven items assessed self-reported anxiety (e.g., “*I was aware of dryness of my mouth*”), and seven items assessed self-reported depression (e.g., “*I could not seem to experience any positive feeling at all*”). Participants responded to each statement on a four-point Likert scale from 0 (“never”) to 3 (“almost always”). Following the procedure indicated by Henry and Crawford (2005), we derived a score for each subscale (depression, stress, and anxiety) by adding up its seven items. These scores were calculated at pre- and post-tests for all participants of both groups, and the higher the score on depression, stress, or anxiety, the stronger the associated severity and symptomatology. Cut-off scores (Lovibond and Lovibond, 1995) have been proposed to define the degree of severity relative to the population, for stress, anxiety, and depression subscales: (i) normal (≤7, ≤3, and ≤4, respectively), (ii) mild (8–9, 4–5, and 5–6, respectively), (iii) moderate (10–12, 6–7, and 7–10, respectively), (iv) severe (13–16, 8–9, and 11–13, respectively), and (v) extremely severe (≥17, ≥10, and ≥14, respectively).

##### Psychological Well-Being

The French version (Trousselard et al., 2016) of the Warwick-Edinburgh Mental Wellbeing Scale (WEMWBS; Tennant et al., 2007) was used to assess psychological well-being during the last week. This self-assessed questionnaire consists of fourteen items (e.g., “*I have been feeling useful*”). Participants responded on a five-point Likert scale from 0 (“none of the time”) to 4 (“all of the time”). By adding up the 14 items as indicated by Tennant et al. (2007), we derived a general psychological well-being score at pre- and post-tests for all participants of both groups. The WEMWBS scale does not have established cut-off points to distinguish between poor, average, and good mental well-being. The recommended approach by the Warwick Medical School is to compare the obtained scores against those of a comparable population to determine how far they fall away from the mean. Since we were specifically interested in French students, we compared the obtained scores with the mean reported in Trousselard et al. (2016) for this population, that is 51.88. A higher score than 51.88 indicates better psychological well-being and, conversely, a lower score indicates poorer psychological well-being.

#### Attentional Abilities

Attentional abilities were assessed with the D2 test (Brickenkamp, 1962). It is a widely used cancelation task to measure attention and concentration abilities (Brickenkamp and Zilmer, 1998). It consists in crossing out targets (the letter “d” with two dashes below and/or above it) while ignoring irrelevant distractors (the letter “d” with one or three dashes, the letter “p” with one, two, or three dashes under and/or below it). In this study, we used the D2-R (Brickenkamp et al., 2016) a computerized version implemented in French (Hogrefe Testsystem, HTS 5, Hogrefe Editions). Its internal consistency was shown for a European population of 18–55 years old, as well as its good test–retest reliability with students (Brickenkamp et al., 2016). At pre- and post-tests, all participants completed 14 successive series including six lines of ten characters. In each series, participants had to cross as many targets as possible in 20 s while scanning row by row from left to right. For each series, three main scores were calculated: (i) rate of processing (TN) corresponding to the Total Number of targets scanned at the final cancelation, (ii) Concentration Capacities (CC) corresponding to TN minus the number of errors of omission (targets not canceled, EO), and minus the number of errors of confusion (distractors canceled, EC): CC=TN−EO−EC, and (iii) accuracy (E%) which corresponds to the percentage of error, i.e., the sum of EC and EO divided by TN: E%=(EO+EC)/TN×100. The individual scores were then summed across the 13 series (systematically excluding the first one usually related to an adjustment period) and standardized to T-scores relative to the European age-specific population. With this quotation, scores are comprised between 25 and 85, with the mean at 50. For a T-score of 50, 50% of Europeans of the same age present a superior score and 50% an inferior one. The farther (higher or lower) the T-score is from 50, the more or less the measured performance is away (superior or inferior) to the average observed performance of the corresponding population.

### Data Analysis

The collected data were processed with MATLAB R2018b (MathWorks, Natick, MA, United States) and Microsoft Excel 2010 (Microsoft Corporation, Impressa systems, Santa Rosa, California, United States) and statistically analyzed using STATISTICA (version 12, StatSoft Inc., Tulsa, OK, United States) software.

For the three variables of the D2-R (TN, CC and E%), and in each group and test, we ensured that none of the analyzed values were outliers (< or > to *mean* ± 3 × *SD*). No outliers were accordingly detected. However, one control group participant was excluded from the D2-R analysis because of a delay in the completion of the post-test.

The scores of the D2-R and psychological variables are reported as means with within-subject correlation-adjusted error bars (*M* ± CI), with CI representing the 95% CI normalized to account for the within-subjects design (Cousineau, 2005; Cousineau et al., 2021). We computed pre-post intervention change scores for all cognitive and psychological measures $(\Delta_{variable}=Score_{posttest}-Score_{pretest}),$ which are reported as means with SD (*M* ± *SD*).

After ensuring normality for all variables, we first analyzed between-group baseline differences (MM group pre-tests compared to control group pre-tests) using *t*-tests or chi-squares comparisons. To reduce the risk of Type 1 error due to multiple comparisons, we used the Holm-Bonferroni correction method (Holm, 1979). The adjusted-value of *p* are accordingly reported for significant *t*-tests. The variables for which statistically significant baseline group differences were found were included in the following analyses as covariates (i.e., sex and all psychological variables, see Cohort Characteristics and Between Groups Baseline Comparisons section). Secondly, we studied between-group differences on pre-post intervention change scores $\left(\Delta_{variable}\right)$ for all the cognitive and psychological measures while controlling for baseline scores differences using a one-way analysis of covariance (ANCOVA; see Cavanagh et al., 2018 for a similar procedure). To ensure that ANCOVA was appropriate for the data, we verified the homogeneity of regression slopes for each outcome variables and the independence of covariates and intervention effect. When the non-interaction assumption of the slopes was not verified, the violator score was transformed to an independent factor and included in a two-way ANCOVA with an interaction term between the studied factors. If effects were significant, Newman–Keuls *post hoc* comparisons were conducted to search for significant pairwise differences. The level of significance was set to 5% (*p* < 0.05). Effect sizes are reported as partial eta square (*η_p_*^2^). For the sake of brevity, only values of significant effects are reported in the Results section.

Internal consistency of psychological measures (stress, depression, anxiety, well-being) was analyzed using McDonald’s omegas in the JASP software (version 0.14.1, JASP Team, Amsterdam, Netherlands). McDonald’s omegas were used in the present study instead of Cronbach’s alphas because the latter have the tendency to over- or underestimate reliability (Dunn et al., 2014).

## Results

### Cohort Characteristics and Between Groups Baseline Comparisons

Stress, anxiety, and depression scores of the final sample (*N* = 76) were in the “mild” category, respectively (*M* ± *SD*) 8.97 ± 5.00, 4.45 ± 3.69, 6.14 ± 4.88. Among them, the percentage of participants with severe to extremely severe scores was 24% for stress, 20% for anxiety, and 18% for depression. The well-being score of the WEMWBS of the final sample was (*M* ± *SD*) 47.30 ± 7.13, with 70% of participants presenting a score below 52.

TN, CC, and E% of the D2-R test of the final sample (*N* = 75) were, respectively, (*M* ± *SD*) 51.59 ± 9.21, 50.69 ± 8.94, and 49.29 ± 8.92.

Regarding between groups characteristics, *t*-tests showed that MM and control groups were not significantly different regarding age [*t*(74) = −0.779, *p* = 0.438], but were significantly different regarding sex [chi-square test: *χ*^2^ (1,76) = 6.408, *p* = 0.011, adjusted *p* = 0.013]. Cohort characteristics, as well as means and confidence intervals for stress, anxiety, and depression, well-being, and attention abilities at the pre-test for each group are presented in Table 1. Compared to the control group, the MM group had statistically significant higher stress [*t*(74) = −3.05, *p* = 0.003, adjusted *p* = 0.010], anxiety [*t*(74) = −3.31, *p* = 0.001, adjusted *p* = 0.007], depression [*t*(74) = −3.56, *p* < 0.001, adjusted *p* = 0.008], and lower well-being scores [*t*(74) = 2.55, *p* = 0.013, adjusted *p* = 0.0.017], but comparable performance in the D2-R test at baseline. Accordingly, sex and all psychological variables were used as covariates in the subsequent analyses of change scores.

**Table 1:** Cohort characteristics and baseline (pre-test) psychological and cognitive measures of both groups.

Variables	Control group (*n* = 38)	MM group (*n* = 38)	Statistics^*^
**Characteristics**			
Number of women	12	23	*χ*^2^(1,76) = 6.41, *p* = **0.011**
Age in years mean (SD)	21.83 (4.13)	22.43 (2.44)	*t*(74) = −0.779, *p* = 0.438
**D2 Mean (CI)**			
TN	50.95 (3.08)	51.34 (2.89)	*t*(73) = −0.23, *p* = 0.819
CC	50.07 (3.02)	51.32 (2.74)	*t*(73) = −0.61, *p* = 0.546
E%	49.10 (2.89)	50.13 (2.8)	*t*(73) = −0.83, *p* = 0.410
**DASS21 Mean (CI)**			
Stress	7.32 (1.55)	10.63 (1.46)	*t*(74) = −3.05, *p* = **0.003**
Anxiety	3.13 (0.91)	5.76 (1.27)	*t*(74) = −3.31, *p* = **0.001**
Depression	4.29 (1.18)	8 (1.66)	*t*(74) = −3.56, *p* = **0.0006**
**WEMWBS Mean (CI)**	49.32 (2)	45.29 (2.36)	*t*(74) = 2.55, *p* = **0.013**

* *Statistically significant value of *p* are highlighted in bold*.

### Adherence to the Online Mindfulness Intervention

The completion rate of the MM group was 79% (*n* = 38/48). Eight among the 10 participants of the MM group who did not complete their post-tests did their pre-tests. Hence, the rate of participants of the MM group who dropped-out from the study was 16.7% (*n* = 8/48).

The participants of the MM group that adhered to the protocol (*n* = 38) followed on average 81.75% (*SD* = 14.1%) of the sessions (representing 14 ± 2 sessions), with 66% among them completing at least 82% of the total number of sessions, and none following less than 59% of the sessions (10/17 sessions).

### Stress, Anxiety, and Depression

**Figure 2:**
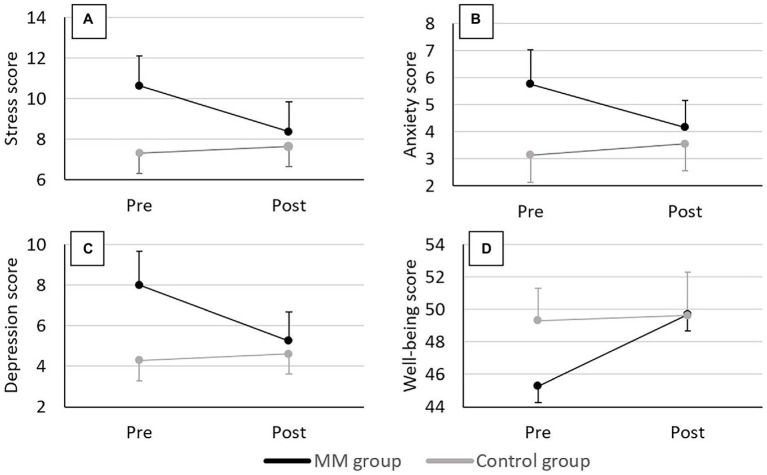
Group scores of the DASS21 and WEMWBS at pre- and post-tests. **(A)** Stress scores, **(B)** Anxiety scores, **(C)** Depression scores, and **(D)** Well-being scores. Error bars represent the normalized 95% CI.

In the final sample, we found good internal consistency (McDonald’s omega) for the stress, anxiety, and depression subscales at pre- (respectively, 0.86, 0.79, and 0.87) and post-tests (respectively, 0.86, 0.71, and 0.90). Group scores of the DASS21’s three subscales at pre- and post-tests are depicted in Figures 2A–C.

For the change score of the stress subscale $(\Delta_{stress}),$ the ANCOVA showed a significant main effect of group after controlling for baseline differences [*F*(1,69) = 7.97, *p* = 0.006, *η_p_*^2^ = 0.10], with a reduction in stress level observed for the MM group $(\Delta_{stress}$ = −2.26 ± 3.58; control group: 0.32 ± 2.77).

**Figure 3:**
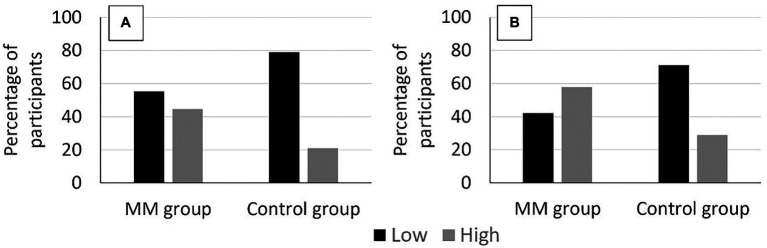
The distribution of participants in the “low” and “high” baseline levels for both the control and the MM groups at pre-test. **(A)** Anxiety scores and **(B)** Depression scores.

For pre-test scores of anxiety and depression, homogeneity of regression slopes assumption was not verified; they were hence transformed to an independent factor with a cut-off of 5 for anxiety and 6 for depression (cf. Materials and Methods section for cut-offs scores). This led to two baseline levels: (i) high anxiety/depression level (grouping moderate, severe, and extremely severe scores), and (ii) low anxiety/depression level (grouping normal and mild scores). The MM and control groups’ distributions under this classification are represented in Figure 3. For the change score of the anxiety subscale $(\Delta_{anxiety}),$ the factorial two-way ANCOVA showed, after controlling for baseline differences, a significant main effect of group [*F*(1,68) = 9.94, *p* = 0.002, *η_p_*^2^ = 0.13] with a reduction in anxiety score observed for the MM group $(\Delta_{anxiety}$ = −1.61 ± 2.95; control group: 0.42 ± 2.06), and a significant main effect of baseline level [*F*(1,68) = 4.29, *p* = 0.042, *η_p_*^2^ = 0.06] with a reduction in anxiety score observed for the participants presenting a high-anxiety level at baseline $(\Delta_{anxiety}$ = −2.28 ± 3.35; low group: 0.24 ± 1.91). It also showed a marginally significant group × baseline score interaction effect [*F*(1,68) = 3.79, *p* = 0.055, *η_p_*^2^ = 0.05]; the *post hoc* decomposition showed significant differences between change scores in MM-high participants relative to the MM-low (*p* < 0.001), control-low (*p* < 0.001), and control-high (*p* = 0.001) participants, with the MM-high showing the greater anxiety reduction $(\Delta_{anxiety}$ = −3.24 ± 3.09, compared to MM-low: −0.29 ± 2.10, control-low: 0.60 ± 1.71 and control-high: −0.25 ± 3.11). For the change score of the depression subscale $(\Delta_{depression}),$ the factorial two-way ANCOVA showed a significant group × baseline score effect after controlling for baseline differences [*F*(1,68) = 5.26, *p* = 0.025, *η_p_*^2^ = 0.07], with the *post hoc* comparisons showing a statistically significant greater reduction in depression scores for the MM-high subgroup $(\Delta_{depression}$ = −4.14 ± 4.50) compared to the MM-low (−0.81 ± 2.01; *p* = 0.005), control-low (0.30 ± 2.51; *p* < 0.001), and control-high (0.36 ± 3.75; *p* = 0.001) sub-groups.

### Well-Being

In the final sample, we found good internal consistency (McDonald’s omega) of the well-being score at pre- (0.82) and post-tests (0.88). Group scores of the WEMWBS at pre- and post-tests are depicted in Figure 2D.

The ANCOVA showed a significant main group effect on pre-post intervention change score of well-being $\left(\Delta_{wellbeing}\right)$ after controlling for baseline differences [*F*(1,69) = 9.03, *p* = 0.004, *η_p_*^2^ = 0.12], with the MM group showing significantly greater improvement in well-being $(\Delta_{wellbeing}$ = 4.39 ± 4.73) than the control group (0.32 ± 6.17).

### Attentional Abilities

After controlling for baseline differences in the relevant variables, the ANCOVA showed no significant effect of the group factor on pre-post intervention change scores of all D2-R’s variables, indicating statistically comparable pre- and post-tests performances in both groups (respectively for the control group and the MM group, $\Delta_{TN}$ = 4.37 ± 5.53/5.03 ± 3.98; $\Delta_{CC}$ = 6.69 ± 5.25/6.89 ± 3.33; $\Delta_{E\%}$ = 5.98 ± 5.89/6.11 ± 7.13).

## Discussion

The main aim of the current study was to evaluate the efficacy of a short online mindfulness intervention in countering the potential psychological distress and attentional deficits in university students constrained to remote learning and lockdown measures during the COVID-19 crisis. Before assessing the outcome of the meditation intervention, we were first interested in confirming whether our participants actually experienced psychological and attentional difficulties.

### Baseline Psychological and Attentional Status

Because at the time of the study no control group or pre-COVID-19 measures could be taken to formally assess the impact of the pandemic, we compared our baseline (pre-tests) results to previous studies and normative values in a similar population. As expected, the participants showed psychological difficulties reflected by their levels of stress, anxiety, and depression with a high prevalence of scores in the extreme to extremely severe categories. In accordance with the findings of Haesebaert et al. (2020) in a 16–29 years old group (*WEMWBS* *M* ± *SD* = 47.80 ± 7.23, and 47.30 ± 7.13 in our study), our results highlighted the significantly poorer well-being score of the French students during the COVID-19 pandemic, compared to previously reported scores (*WEMWBS M* ± *SD* = 51.88 ± 6.87 in Trousselard et al., 2016; *t*-test comparison using *t*-values computed from means and SD reported in both studies: *t*(149) = 4.019, *p* < 0.001). These worrying figures in the psychological domain support the urgent need of identifying preventive and interventional strategies to help university students cope with the additional psychological distress they are facing during the pandemic, such as the online MMI investigated in this study. However, the observed scores for the D2-R test reflecting attentional abilities were situated in the average range of the age-specific European population (T-score around 50), indicating no substantial deficiency in this area. This finding corroborates the choice of keeping the students academically active. Being enrolled, even remotely, in a study program seems to give enough cognitive stimulation so the psychological distress would not compromise the attentional performance of students.

### Feasibility of the Online Mindfulness Intervention

Compared to previous online MMI in students, our intervention showed a high completion rate of 79% (58% in Cavanagh et al., 2018, 71% in Danilewitz et al., 2018), and a satisfying adherence rate to the intervention, with participants completing on average 82% of the sessions (50% in Noone and Hogan, 2018; 95% in González-garcía and López, 2021). Our results are similar to the ones of Haliwa et al., 2021 (90% of completion rate) in undergraduate students using a mobile mindfulness application which offered 5–15 min guided exercises over 10 days. Overall, it appears that guided-MM audio sessions freely available at the end of the day (20 min during weekdays and 10 min during the weekend) and coupled with a motivational follow-up is a feasible and suitable intervention for University students, even during unusually straining times. In contrast with González-garcía and López (2021), the feasibility of our protocol was shown among students who were enrolled in different postgraduate or masters years of different majors (Engineering, Sports Sciences, Education Sciences).

### Efficacy of the Online Mindfulness Intervention on Mental Health and Attentional Abilities

We were expecting our intervention to reduce the MM group’s stress, anxiety, and depression levels, while enhancing their psychological well-being and attentional abilities.

Our results confirmed the expected benefits of MM practice on the psychological level. The observed reduction in stress, anxiety, and depression and the enhanced psychological well-being adds on to the previously reported benefits in general population during the COVID-19 health crisis (Khandelwal, 2020; Lim et al., 2020; Matiz et al., 2020; Sanilevici et al., 2021), and extends those observed in university students outside this particularly straining context (Spadaro and Hunker, 2016; Cavanagh et al., 2018; El Morr et al., 2020; Moore et al., 2020). It also confirms the reduction in anxiety and depression suggested by González-García and López’ study that used different measures and lacked a control group (González-garcía and López, 2021). The current study supports the efficacy of MM in modulating the negative psychological states inherent to the ongoing pandemic (Khandelwal, 2020), even when practiced briefly online. Furthermore, our results suggested that MM benefits were especially robust for the participants presenting high baseline anxiety and depression levels. Hence, it seems that brief online MMI could be more helpful for those who suffer from a poorer mental health. This conclusion should be tempered since the observed results were based on statistical interactions with a small effect size. Nevertheless, converging evidence was reported elsewhere (Schreiner and Malcolm, 2008). In their 2008 study, Schreiner and Malcolm also used the DASS-21 and found that participants with severe emotional difficulties at baseline demonstrated the most notable improvement after a 10-week MM program. MM practice could hence play a role in normalizing emotional states. As emotional distress is a result of non-acceptance of one’s emotions rather than the emotion itself (Barlow et al., 2004), by developing self-awareness, acceptance, and non-reactivity MM practice could improve mental health. This could be linked to the MM-related increase in parasympathetic activity and decrease in sympathetic one (for review see Vago and Silbersweig, 2012), which reduces the physiological response to poor mental health. Conceptually, it is argued that developing trait mindfulness through mindfulness practice enables the person to better tolerate their affect and eventually be liberated from its negative impact (Bishop et al., 2004; Shapiro et al., 2006).

On the cognitive level, contrary to our expectations, our results showed that attentional improvement cannot be attributed to the studied MMI. Notwithstanding, the expected attentional difficulties were also not found at baseline to start with. So, it might not be surprising that the tested short intervention did not produce a noticeable enhancement in attentional abilities in already good performing young participants. To this end, a longer intervention may be needed, as shown for instance by Jha et al. (2007) after 5 weeks of MM training in students. Finally, the remote learning experience might have actually offered an opportunity to students to maintain their cognitive faculties. This may be related to cognitive stimulation and to the mitigation of the lockdown-imposed social isolation. A total academic interruption scenario may have had greater impacts on cognitive performance. According to Ingram et al., 2021, social isolation can cause cognitive decline regardless of age. So it is a possibility that the online daily interactions with teachers and other students offered further cognitive protection.

Overall, although the tested mindfulness intervention did not enhance the attentional abilities in already good performing students, it did promote their mental health that seemed to be significantly compromised by the pandemic context.

### Conclusions, Limitations, and Future Directions

This study confirms the mental health challenge imposed by the COVID-19 lockdown on university students. It also offers additional evidence on the feasibility and efficacy of mindfulness-based interventions in helping students to cope with mental distress during psychologically straining periods, like the current pandemic, and potentially a similar future crisis. Indeed, the adherence to the intervention was very promising, and despite the relatively short period of practice, positive psychological effects were observed on stress, anxiety, depression and well-being.

These encouraging findings come however with some limitations that need to be addressed in future work. One limitation is the absence of an active control group. This raises the possibility of non-specific aspects to the mindfulness intervention contributing to the observed effects, and that the (non-blind) control group would be biased toward not achieving better in post-tests. As suggested by Davidson and Kaszniak (2015), a more rigorous, but undeniably more challenging approach, for a future study would be to include several control groups, designed to rule out alternative explanatory mechanisms of the expected effects, within a “dual-blind” design. The present study used also a non-stratified randomization procedure leading eventually to significant differences at baseline between the experimental and control groups. Although, this was controlled for in the statistical analysis, it would be interesting to specifically investigate in balanced groups the effects of baseline score levels and performance on the outcomes of the studied MMI. This would help in providing more individual-specific practice recommendations. It is also worth mentioning that the per-protocol analysis that was used here, wherein drop-outs are excluded, may lead to an overestimation of the benefits in real population. Unfortunately, we had no post-tests data for the participants who did not follow or discontinued the protocol to satisfyingly conduct an intention to treat analysis. Nevertheless, most of the possible biases that can result from the per-protocol analysis (Ranganathan et al., 2016), were either inapplicable or controlled for in our study. Finally, an inherent limitation of online experimentation is the challenge of objectively monitoring the participants during the testing and the intervention sessions. Indeed, although the participants were given detailed instructions and were reminded of them throughout the tests and the sessions, we cannot be sure how respectful and punctual they were in reality. Ideally, for future research, pre- and post-tests must be realized in laboratory-controlled conditions with the presence of the experimenter. Unfortunately, this was not possible due the health restrictions imposed at the time of the present study. The pandemic context also constrained our sample size and the duration of the intervention. Testing a larger cohort with longer online MMI in future research could potentially induce attentional effects that we might have missed here, and allow the specific investigation of the relationship between cognitive abilities and mental health.

## Data Availability Statement

The raw data supporting the conclusions of this article will be made available by the authors, without undue reservation.

## Ethics Statement

The studies involving human participants were reviewed and approved by the Ethics Committee for Research in Science and Techniques of Physical and Sports Activities (CER STAPS no IRB00012476-2020-25-11-70). The patients/participants provided their written informed consent to participate in this study.

## Author Contributions

All authors participated in conceiving the study design. RS-M and LD-R conducted the experiment, collected the data, and analyzed the results. All authors contributed to the article and approved the submitted version.

## Conflict of Interest

The authors declare that the research was conducted in the absence of any commercial or financial relationships that could be construed as a potential conflict of interest.

## Publisher’s Note

All claims expressed in this article are solely those of the authors and do not necessarily represent those of their affiliated organizations, or those of the publisher, the editors and the reviewers. Any product that may be evaluated in this article, or claim that may be made by its manufacturer, is not guaranteed or endorsed by the publisher.
