# Supplementary material for: Online Mindfulness Intervention, Mental Health and Attentional Abilities: A Randomized Controlled Trial in University Students During COVID-19 Lockdown
Source: Front Psychol. 2022 Jul 7;13:889807. doi: 10.3389/fpsyg.2022.889807 (PMC9301203; doi:10.3389/fpsyg.2022.889807)
Supplement: Supplementary file 1 [file Table_1.DOCX]

Supplementary Material

Supplementary Table 1. Summary of the organization and the content of the 17 mindfulness meditation sessions.

| **Session**  ***(Duration in minutes)*** | **General topic of the MM exercise** | **Theme of the introduction**  ***(Duration in minutes)*** | **Exercise instructions**  ***(Exercise duration in minutes)*** | **Conclusion / Recommendations**  ***(Duration in minutes)*** |
| --- | --- | --- | --- | --- |
| Session 1  *12:38* | Mindfulness of the breath | Presentation of the instructor and their personal experience with MM. *(1:20)* | Count the breaths from 1 to 10, then 1 to 20. If distractions occur, start over from 1. Do not change intentionally the breathing rate. *(10:12)* | Encouragements to continue practice, with an overview on MM benefits in daily life. *(1:06)* |
| Session 2  *21:12* | Mindfulness of the breath | Brief presentation of the stereotypes on MM, followed by an overview of its main benefits, and finally an introduction of the technique used in this session: internally noticing the thoughts. *(1:50)* | Focus attention on breathing while noticing every disturbing thought. Consider these thoughts as clouds passing in the sky, and then draw your focus back on the breath. *(18:11)* | Daily awareness of disruptive thoughts. *(1:11)* |
| Session 3  *21:56* | Mindfulness of the breath | Origin of the technique used in this session: Thích Nhât Hanh.  Presentation of a Buddhist monastery, the Village of the Plum trees. *(1:15)* | For each breath, state mentally “I am breathing in, I know that I am breathing in” followed by “I am breathing out, I know that I am breathing out”. *(19:20)* | In daily life, when necessary, try focusing on your breath to draw your attention to the present moment. *(1:21)* |
| Session 4  *21:36* | Mindfulness of the breath | Origin of the technique used in this session: Goenka.  Description of the instructor’s 10-day retreat experience. *(1:14)* | Focus on the sensations arising around the nostrils during breathing. *(19:08)* | Attention is a precious resource overloaded by a busy life style and new technologies. MM practice enables us to train our attention to be less in an automatic pilot mode and be more aware of the present. *(1:14)* |
| Session 5  *20:41* | Mindfulness of the breath and acceptance | Definition of acceptation. *(1:22)* | Focus on the present moment (sensations, thoughts) and on the breath without analysing or reacting.  *(18:06)* | In daily life, try to accept pleasant and unpleasant events. *(1:13)* |
| Session 6  *12:12* | Acceptance | How to develop acceptation in daily life. *(1:35)* | Focus on breathing (rhythm, duration, break time between inspiration et expiration) and accept it as it is. Then, explore sensations of breathing in the entire body. *(10:05)* | In daily life, observe breathing with curiosity at different times and different places. *(0:32)* |
| Session 7  *13:10* | Acceptance | Presentation of the objective of the session: acceptation of body sensations, even unpleasant and painful ones. *(0:35)* | Focus on unpleasant body sensations or on a painful zone. Do not react or judge your feelings, bring pleasure to this part of the body. *(12:07)* | In daily life, observe painful body sensations with curiosity at different times and different places. *(0:28)* |
| Session 8  *20:26* | Mindfulness of the breath | Origin of the technique used in this session: John Kabat-Zinn. *(1:12)* | Focus on breathing while being aware of all the arising body sensations. *(18:18)* | Explanations of why breathing is an essential tool in MM practice. *(0:56)* |
| Session 9  *19:58* | Positive emotions:  Love | The importance of self-love.  Presentation of a new technique used in all “Positive emotions” exercises: visualisation. *(0:39)* | Remember with details a situation procuring self-love. Have a full sense of this self-love and be aware of the resulting body sensations. Welcome the positive feelings and sensations. *(17:44)* | Daily, practice loving kindness towards loving and aggressive persons. *(1:35)* |
| Session 10  *20:38* | Positive emotions:  Gratitude | Definition of gratitude. *(1:00)* | Same instructions as in session 9 with a situation procuring gratitude. *(18:34)* | Before the next session, thank someone who has done something good.  Daily, before going to bed, try to note 3 things for which you felt grateful during the day.  *(1:04)* |
| Session 11  *20:27* | Positive emotions:  Amazement | Definition of amazement. *(0:57)* | Same instructions as in session 9 with a situation procuring amazement. *(18:27)* | Before the next session, observe the world with the inner child and amazement about simple things. *(1:03)* |
| Session 12  *20:22* | Positive emotions: Enthusiasm | Definition Enthusiasm. *(1:23)* | Same instructions as in session 9 but with a situation procuring enthusiasm. *(18:09)* | Before the next session, talk about dreams and passions to at least one person and share the enthusiasm it procures. *(0:50)* |
| Session 13  *11:01* | Acceptance | How being in the present moment enables to accept difficult emotions. *(0:31)* | Focus on an actual difficult situation or a life problem. Be aware of the body sensations caused by this latter, especially in the throat, the chest and the stomach. Put a word on the main sensation.  *(10:11)* | In daily life, observe difficult emotions and sensations caused by it in different time and place. *(0:19)* |
| Session 14  *11:15* | Acceptance | The importance of noticing emotions and sensations to develop acceptance. *(0:28)* | Focus on a current difficult situation. In the presence of self-judgment, take note of it. Develop self-compassion by having a tender gesture toward oneself. *(10:42)* | At the end of each session, try to note the experienced feelings. *(0:05)* |
| Session 15  *20:37* | Positive emotions: Affection | Definition of affection. *(1:46)* | Same instructions as in session 9 with a situation procuring affection. *(17:59)* | In daily life, share affectionate moments with the entourage. *(0:52)* |
| Session 16  *20:05* | Positive emotions:  Hope | Explanation of the important role of negative emotions in daily life.  Definition of hope. *(1:30)* | Same instructions as in session 9 with a situation procuring hope. *(17:40)* | Surround oneself by optimist persons.  Until the next session, reconnect oneself 2 times to the hope felt in the session of the day. *(0:55)* |
| Session 17  *19:13* | Positive emotions: Happiness | Definition of happiness. *(1:06)* | Same instructions as in session 9 with a situation procuring happiness. *(17:58)* | Conclusion of the intervention. *(0:09)* |
